# Investigating retrotransposon‐derived eccDNA as a source of innate immune activation in tauopathy

**DOI:** 10.1002/alz.094614

**Published:** 2025-01-09

**Authors:** Morgan Elizabeth Lambert, Paulino Ramirez, Wenyan Sun, Bess Frost

**Affiliations:** ^1^ Center for Alzheimer’s Disease Research, Providence, RI USA; ^2^ Robert J. and Nancy D. Carney Institute for Brain Science, Providence, RI USA; ^3^ Brown University, Providence, RI USA; ^4^ University of Missouri‐Kansas City, Kansas City, MO USA

## Abstract

**Background:**

Retrotransposon‐derived extrachromosomal circular DNA (eccDNA) was extracted and sequenced from brains with Alzheimer’s disease, progressive supranuclear palsy, or healthy controls. Retrotransposon‐derived DNA was visualized outside of the nucleus in these phenotypes with phospho‐STING. *Drosophila* were used as a model to study extranuclear retrotransposon DNA.

**Method:**

DNA was extracted from post‐mortem human patients with AD (n = 6), PSP (FTD) (n = 6), or no pathology (n = 6). The DNA was treated with exonucleases to degrade linear DNA and the circular DNA was isolated. Post‐mortem cryosections of AD, PSP, or healthy control brains were used for fluorescent in‐situ hybridization (FISH) of retrotransposon sequences. Co‐immunofluorescence of phospho‐STING was performed on FISH tissue. *Drosophila* brains were also used for FISH of retrotransposons. Digital qPCR was used to measure copy number of retrotransposons in human and fly tissue.

**Result:**

Preliminary data suggests an increase in phospho‐STING in AD and PSP brains compared to controls. Preliminary data also suggests an enrichment of retrotransposon DNA in circular DNA sequencing in tau pathology compared to controls.

**Conclusion:**

Retrotransposon‐derived eccDNA may be an activator of the cGAS‐STING pathway in AD and PSP.

